# A simulation approach to assessing environmental risk of sound exposure to marine mammals

**DOI:** 10.1002/ece3.2699

**Published:** 2017-02-28

**Authors:** Carl R. Donovan, Catriona M. Harris, Lorenzo Milazzo, John Harwood, Laura Marshall, Rob Williams

**Affiliations:** ^1^ Centre for Research into Ecological and Environmental ResearchThe ObservatoryUniversity of St AndrewSt AndrewsUK; ^2^ Imperial College LondonNHLI, St. Mary's Campus Norfolk PlaceLondonUK; ^3^ Sea Mammal Research UnitScottish Oceans InstituteUniversity of St AndrewsSt AndrewsUK

**Keywords:** agent‐based models, gray seal, harbor porpoise, risk assessment, underwater sound

## Abstract

Intense underwater sounds caused by military sonar, seismic surveys, and pile driving can harm acoustically sensitive marine mammals. Many jurisdictions require such activities to undergo marine mammal impact assessments to guide mitigation. However, the ability to assess impacts in a rigorous, quantitative way is hindered by large knowledge gaps concerning hearing ability, sensitivity, and behavioral responses to noise exposure. We describe a simulation‐based framework, called SAFESIMM (Statistical Algorithms For Estimating the Sonar Influence on Marine Megafauna), that can be used to calculate the numbers of agents (animals) likely to be affected by intense underwater sounds. We illustrate the simulation framework using two species that are likely to be affected by marine renewable energy developments in UK waters: gray seal (*Halichoerus grypus*) and harbor porpoise (*Phocoena phocoena*). We investigate three sources of uncertainty: How sound energy is perceived by agents with differing hearing abilities; how agents move in response to noise (i.e., the strength and directionality of their evasive movements); and the way in which these responses may interact with longer term constraints on agent movement. The estimate of received sound exposure level (SEL) is influenced most strongly by the weighting function used to account for the specie's presumed hearing ability. Strongly directional movement away from the sound source can cause modest reductions (~5 dB) in SEL over the short term (periods of less than 10 days). Beyond 10 days, the way in which agents respond to noise exposure has little or no effect on SEL, unless their movements are constrained by natural boundaries. Most experimental studies of noise impacts have been short‐term. However, data are needed on long‐term effects because uncertainty about predicted SELs accumulates over time. *Synthesis and applications*. Simulation frameworks offer a powerful way to explore, understand, and estimate effects of cumulative sound exposure on marine mammals and to quantify associated levels of uncertainty. However, they can often require subjective decisions that have important consequences for management recommendations, and the basis for these decisions must be clearly described.

## 1 Introduction

A series of high‐profile strandings of beaked whales following naval sonar exercises in the late 20th century (reviewed in Jepson et al. (2003)) drew public attention to the potential effects of intense anthropogenic ocean noise on marine organisms and convinced many scientists and policymakers that ocean noise is a pervasive, globally important environmental issue. In the subsequent decades, tremendous progress has been made in understanding the responses of sensitive species to particularly aversive sounds (Tyack et al., 2011). Regulatory agencies around the world are routinely required to approve or deny permit applications for industrial activities in important marine mammal habitats that may generate impulsive sound levels that are comparable to those produced by sonars. The two main activities that fall into this category are pile driving (Bailey et al., 2010) and the use of airguns in offshore oil and gas exploration (McCauley, Fewtrell, & Popper, 2003).

We developed a simulation framework, which we have called “SAFESIMM” (Statistical Algorithms For Estimating the Sonar Influence on Marine Megafauna), that uses agent‐based models to quantify the extent to which marine mammals may be affected by proposed noise‐generating activities. Here, we describe that framework and explore the sensitivity of its predictions to uncertainty relating to different model components. Our framework is one of a small number of risk assessment tools available to the scientific, ocean business, and regulatory communities. Other published examples include 3 MB (Houser, 2006), AIM (Frankel, Ellison, & Buchanan, 2002) and ESME (Shyu & Hillson, 2006). All of these statistical tools have to solve a common set of problems, which we list below. We describe the statistical derivation of SAFESIMM and similar risk assessment frameworks, investigate which aspects of these frameworks are most vulnerable to knowledge gaps, and identify priority research areas.

Two key lessons have emerged from the development of management procedures that set sustainable limits to direct and indirect lethal takes of marine mammals. First, any scientific advice must be robust to uncertainty (Harwood & Stokes, 2003; Taylor, Wade, de Master, & Barlow, 2000). For example, marine mammal abundance estimates generally suffer from low precision, so marine mammal scientists have been early adopters of precautionary approaches to management (Taylor, Martinez, Gerrodette, Barlow, & Hrovat, 2007; Wade, 1998). Secondly, a formal and well‐specified management strategy evaluation process is needed to adapt to new information (Cooke, 1999; Punt & Donovan, 2007). SAFESIMM satisfies the first criterion because it is constructed in a modular way to account for uncertainty in all of the components of the simulations. However, although SAFESIMM and similar frameworks have been used extensively by industry and regulators to explore effects of noise‐generating activities on a variety of marine mammal species, their performance has not previously been subjected to the kind of statistical scrutiny that forms the core of management strategy evaluation. This requires a transparent exploration of the sensitivity of model outputs to misspecification and uncertainty in key inputs.

A useful description of a quantitative risk assessment was provided by Zacharias and Gregr (2005). The authors partition risk into two components: *sensitivity*, which is the degree to which organisms respond to a stressor (i.e., deviations in environmental conditions beyond the expected range); and *vulnerability*, which is the probability that an organism will be exposed to a stressor to which it is sensitive. For our purposes, a marine mammal's sensitivity to sound has to do with features of the sound exposure (e.g., received level in different frequency bands and duration) and the biology of the animal (e.g., the species’ dose–response curve, its hearing ability (audiogram), the ecological context in which the stressor occurs (Ellison, Southall, Clark, & Frankel, 2011; Williams, Lusseau, & Hammond, 2006), and the evasive tactics or movement patterns it exhibits in response to exposure). Vulnerability is a function of marine mammal distribution and abundance in space and time (with associated measures of uncertainty), and the noise levels experienced by each individual. The latter are determined by propagation models that predict received sound levels, depending on source levels, peak frequencies and bathymetry, and each individual's response to the received sound levels.

Industrial developments that generate high‐amplitude noise within important marine mammal habitats generally have to comply with country‐specific policies that require an assessment of the harm likely to result from those activities. These assessments may be at the individual or population level and allow managers, regulators, and decision makers to evaluate whether such levels of risk are acceptable. While the details of those policies vary from country to country (Horowitz & Jasny, 2007), they generally include an overarching requirement for an estimate of the number of individuals of a given species that are expected to experience received noise levels high enough to cause behavioral disturbance or injury, namely a permanent or temporary loss of hearing sensitivity (e.g., a permanent threshold shift, “PTS,” or a temporary threshold shift, “TTS”; Southall et al., 2007). That number, referred to as a “take” under US policies, along with consideration of the population's conservation status forms the basis of a decision on whether to authorize the activity. Such authorizations are generally subject to conditions that require the proponent to mitigate harm wherever feasible. Although most national policies require estimates of take in terms of individual animals exposed, newer analytical methods aim to quantify potential impacts to populations (Harwood, King, Schick, Donovan, & Booth, 2014; New et al., 2014) or important habitats (Erbe, MacGillivray, & Williams, 2012). Our focus is at the level of individuals.

Although national policies are spelled out in terms of overarching objectives, implementation relies on considerable discretion from regulatory agencies. Taken as a whole, the process of quantifying risk associated with marine mammals and noise‐generating activities involves highly technical and interdisciplinary discussions, with aspects of the assessment partitioned and considered separately by experts in the fields of statistical and acoustic modeling, marine biology, physiology, marine spatial planning, and quantitative risk assessment (Harwood, 2000). Given the uncertainty inherent in estimating the abundance, distribution and movements of marine mammals, sound field propagation, and behavioral and physiological responses of marine mammals to noise, the field of noise impact assessments lends itself to probabilistic approaches to simulating all of these sources of variability. In practice, the physical acoustics literature often ignores uncertainty in sound field propagation modeling (Erbe et al., 2012).

As a result of the current compartmentalization of specialties involved in assessing the risk to marine organisms from anthropogenic noise, it would be easy for regulators to miss, or misunderstand, some of the assumptions that must be made during these assessments. The offshore renewables industry, with its associated noise production from pile‐driving activities, is large and growing (Gill, 2005), and many regions of the world's oceans are dominated by seismic survey noise (Gordon et al., 2003). In our view, the sheer number of noise‐generating activities being evaluated and permitted each year around the globe creates a need to evaluate the performance of the risk assessment tools currently in use and to make practical suggestions about the best way to provide robust scientific advice that takes account of uncertainty associated with these assessments.

We originally developed SAFESIMM to quantify impacts of naval sonar use on marine mammals, and as such, the methodology has been scrutinized by the naval community (Mollett et al., 2009). More recently, SAFESIMM has been used to assess the potential effects of noise associated with offshore renewable energy construction in the UK. Here, we undertake a formal evaluation of the performance and strengths and weaknesses of agent‐based simulation tools using SAFESIMM as an example framework. We document the assumptions underlying our simulation framework and identify situations when its predictions may be unreliable. These tools were originally designed to understand the impacts of short‐term tactical sonar exercises, carried out over hours or days, rather than activities that may take place over weeks, months, or years. Given the central role that such tools play in the production of marine mammal impact assessments (MMIAs), it is important to explore the consequences of different parameterizations and model assumptions. This will allow regulators to better understand the basis for the MMIAs and have more confidence in their own permitting decisions. For illustrative purposes, we use PTS as the response variable of interest, but risk tolerance is a policy decision. Managers may wish to minimize TTS or the number of behavioral disturbance events, in which case simulation approaches like SAFESIMM can be easily adapted to track other noise exposure metrics.

## 2 Methods

SAFESIMM (Donovan, Harris, Harwood, & Milazzo, 2012) was developed in conjunction with BAE Systems Insyte Ltd. from 2005 and served as the template for their Environmental Risk Mitigation Capability (ERMC) software (Mollett et al., 2009). All code was written in the statistical programming environment R (R Development Core Team, 2011).

**Figure 1:**
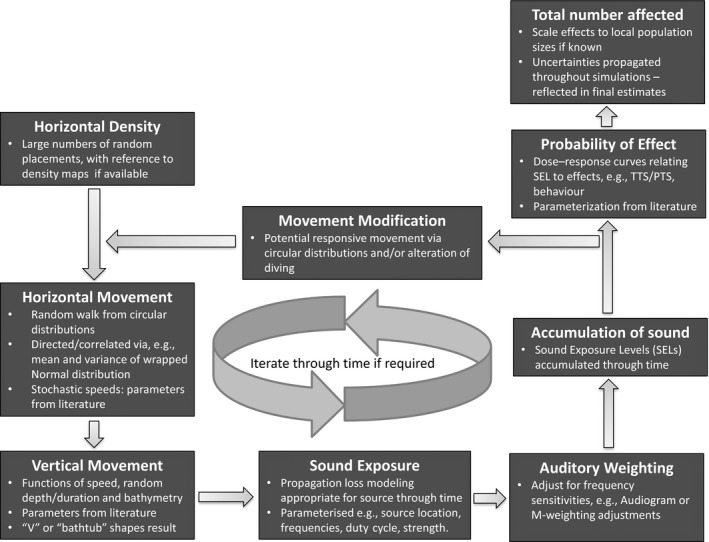
The modular nature of SAFESIMM

We provide an overview of the agent‐based approach (Bonabeau, 2002) used within SAFESIMM and describe the individual components of the framework. We then describe a set of scenarios that were used to test the sensitivity of the predictions made by SAFESIMM to key assumptions. The modular structure of SAFESIMM is shown in Figure 1, and the inputs required by each module are described in Table 1.

**Table 1:** The modules of SAFESIMM as they contribute to describing the vulnerability and sensitivity of marine mammals to sound exposure, and the required inputs for the modules

	SAFESIMM module	Required inputs
Vulnerability (probability that marine mammals will be exposed to noise to which they are sensitive)	Horizontal density	Estimated/predicted number of animals (with measure of uncertainty, e.g., CVs) by space and time
	Horizontal movement, vertical movement, movement modification	Dive depth, dive duration, swim speed, surface time, group size, bathymetry, and coastline
	Sound exposure	SPL in dB. Typically a library of precalculated sound fields covering the extent of the scenario.
	Accumulation of sound	Duty cycles, timings and frequencies for the scenario. Linked to specific sound fields in the library and generate sets of sound exposure histories (SEL through time)
Sensitivity (degree to which marine mammals will respond to noise)	Horizontal movement, vertical movement, movement modification	Dive depth, dive duration, swim speed, surface time, group size, movement in response to sound, bathymetry, and coastline
	Auditory weighting	Audiograms (A‐weighting), M‐weighting functions
	Probability of effect	Dose–response curve or threshold values for response (TTS/PTS or behavioral)

The movement of thousands of agents representing dozens of species is tracked through time within each simulation, and received sound levels (RLs) for each agent are recorded at each time step by reference to the input sound field. These RLs are then weighted to account for the hearing sensitivities of the different species at the relevant frequency, and the resulting sound exposure is accumulated over time. These accumulated, weighted SELs are then used as input to dose–response relationships to determine the probability that an agent will experience a physiological effect (i.e., PTS or TTS) or exhibit a behavioral response (e.g. Moretti et al. 2014, Williams, Erbe, Ashe, Beerman, & Smith, 2014). At the end of the simulation process, the sound histories for each agent and the number of physical and behavioral effects they experienced are summarized.

### 2.1 Horizontal density

Density data, with associated measures of uncertainty, are required by the horizontal density module (Figure 1, Table 1) to allow agents to be distributed through a sound field in a realistic way. The framework can accept gridded density data at any resolution with density expressed as animals per km^2^, and an associated coefficient of variation (CV). The density data used in the scenarios described below were generated based on the results of modeling which combined available survey data with an index of relative environmental suitability (RES; Kaschner, Watson, Trites, & Pauly, 2006). This allowed us to extrapolate density estimates to areas with no survey data. However, any suitable species density or abundance map can be used to seed the simulations.

### 2.2 Horizontal and vertical movement

SAFESIMM models the “natural” movement of agents in both horizontal and vertical planes, and their responses to acoustic disturbance. These responsive movements are modeled by modifying the natural patterns of movement. For example, each species has diving and swimming characteristics, such as maximum dive depths, dive durations, and typical and maximum swim speeds. These can be thought of as parameters governing a directed random walk that is used to simulate movement. Some species are reported to cease diving in the presence of acoustic disturbance, and others may exhibit fleeing behaviors. Although these processes are generally poorly understood, key parameters of the movement model can be modified to reflect the latest state of knowledge.

We reviewed the literature on the natural and responsive movements of the 115 marine mammal species that can be modeled using SAFESIMM and compiled a database of relevant parameter values and functions. These parameters include dive depth, dive duration, swim speed, surface time, group size, and whether or not agents are known to respond to noise. The responsive movement parts of the database include parameters that govern functions for dive shapes and dose–response. The database also contains information on audiograms and M‐weighting functions. If no data were found for a species and field, a value was inferred from the most closely related species in the database.

Bathymetric data for the area of interest are also required, so that the movements of individual agents can be related to the physical environment. This ensures that agents do not dive below the seafloor, or swim onto land.

### 2.3 Sound exposure

The RL for each agent at each time step is calculated using an estimated sound field specific to the properties of the sound and the area in which the sound source is located. These sound fields are generated using sound propagation models that calculate the loss of sound energy as it travels away from the source. Sound propagation through water is dependent on source level and sound frequency, plus a number of physical factors, for example water depth and temperature. The framework is flexible as regards propagation loss models, and the agents simply call for a predicted sound level at a particular point at a particular time.

Industrial activities are rarely continuous, and so the sound exposure module has a built‐in duty cycle that determines the frequency with which the sound source is active, and this determines the amount of time that agents are actually exposed to sound.

### 2.4 Auditory weightings

**Figure 2:**
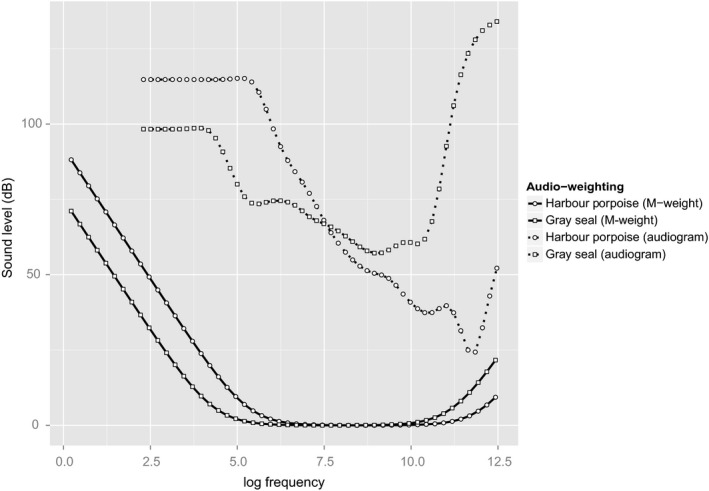
Southall et al.'s (2007) M‐weighting functions for the functional groups that include gray seal and harbor porpoise and corresponding audiogram weightings (A‐weightings). Sound levels are dB re 1 μPa2/s

Once the RL for each individual agent at each time step has been calculated, it is weighted to allow for the species’ hearing sensitivities at given frequencies. Two auditory weighting schemes are supported in the SAFESIMM: one derived from the species’ audiogram (the measured or inferred hearing thresholds plotted over a range of frequencies), referred to hereafter as an A‐weighting (“A” for audiogram); and one derived from the M‐weightings developed by Southall et al. (2007). To determine these weighting, Southall et al. (2007) classified all marine mammal species into five functional groups, on the basis of their phylogeny, and their measured or estimated hearing characteristics. These groups are: low‐frequency cetaceans (baleen whales), medium‐frequency cetaceans (beaked whales and most dolphins), high‐frequency cetaceans (porpoises, freshwater dolphins, and dolphins in the genus *Cephalorhynchus*), pinnipeds (seals and sea lions) in water, and pinnipeds in air. M‐weightings are markedly different from, and simpler than, the A‐weightings for our species of interest (Figure 2).

### 2.5 Probability of effect

The probability that an agent will respond to the weighted SEL that it is estimated to receive over a particular time interval can be determined using a simple threshold, or a dose–response relationship. Southall et al. (2007) recommend different thresholds for permanent threshold shift (PTS) for each functional group, and for pulsed and nonpulsed sound. For the simulations presented here, we adopt the simple thresholds of Southall et al. (2007), or Heathershaw et al. (2001). However, SAFESIMM typically uses a dose–response relationship for PTS that is derived from similar data to that used by Southall et al. (2007) for their thresholds. It is based on the results of experimental studies of a range of marine mammal species summarized in Finneran, Carder, Schlundt, and Ridgway (2005). These predict that statistically significant temporary threshold shift (TTS) begins to occur at an SEL of 195 dB re 1 μPa^2^/s. This equates to a predicted probability of TTS of 0.18–0.19 based on an approximation of the fitted curve reported in Finneran et al. (2005).

### 2.6 Model outputs

The current summary outputs provided by SAFESIMM are the probability (by species) that any agent will experience PTS and the expected number of agents within each species that are expected to experience TTS. This information can be summarized for an entire area or displayed at the spatial resolution of the input data, allowing areas of high and low risk to be identified.

All density estimates held in the internal database have an estimate of uncertainty associated with them. These uncertainties, together with the uncertainty associated with the other parameters used in the simulation process, allow confidence intervals to be provided for any outputs.

### 2.7 Simulations/case studies

Three sets of scenarios were considered, in which agents were exposed to a modeled sound field based on a 1‐kHz nonpulsed sound source with a source strength of 240 dB re 1 μPa2/s and a 10% duty cycle over periods ranging from 1 hour to 10 days. All simulations were based on 10,000 agents, 15 log(R) propagation loss models, and a uniform 50‐m bathymetry. Species’ distributions, speeds, and diving characteristics were from sources described previously.

1. *Auditory weighting*. We calculated SELs for gray seals and harbor porpoises using both A‐ and M‐weighting. At this frequency, the M‐weighting for both species is effectively zero.
2. *Responsive movement*. For gray seals, SELs were calculated under different assumed levels of avoidance, ranging from no response to very marked avoidance. Movement was modeled as a directed random walk (in the statistical sense) away from the source. A wrapped normal distribution was chosen for computational speed (Agostinelli, 2012; Jammalamadaka & Sengupta, 2001). Two parameters (mean and variance) governed directionality and dictated how similar sequential random draws would be. A high variance results in movement that is erratic: effectively a directionless random walk. As the variance is decreased, movement becomes more directed. In the extreme case of zero variance, every draw from the distribution involves continual movement in the same direction. The standard deviations (SD) used were 10, 1, 0.5, 0.1, and 0.05, going from directionless movement to directed fleeing.
3. *Constrained movement*. In these simulations, we compared situations in which the movement of agents was effectively unconstrained for up to 10 days, with those in which there was a hard boundary preventing movement beyond 75 or 100 km. These simulations were carried out for gray seals, using M‐weighting, and responsive movement variances of 0.5 and 10.

## 3 Results

### 3.1 Auditory weighting

The number of agents that might experience PTS was calculated using different threshold values for the M‐ and A‐weighting schemes. We used the threshold recommended by Southall et al. (2007) with the M‐weighting scheme and an “audiogram appropriate” threshold proposed by Heathershaw, Ward, and David (2001) with the A‐weighting scheme—the threshold being 95 dB above the threshold of hearing.

**Figure 3:**
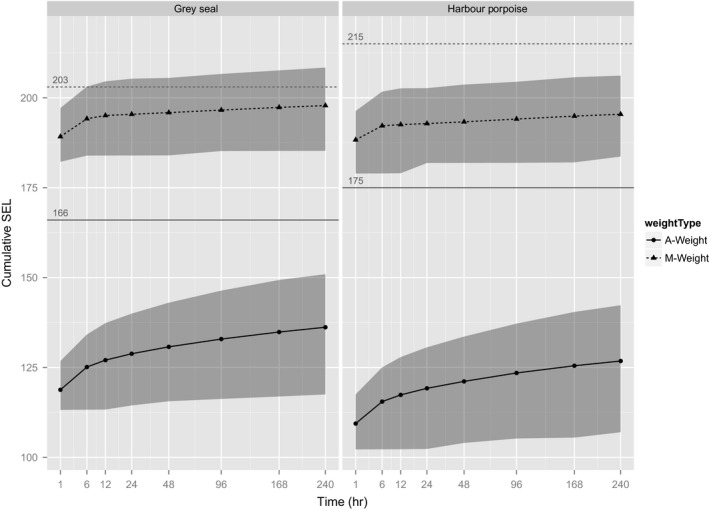
Comparing the effect of M‐ versus A‐weightings on predicted mean SELs for two species over time—M‐weightings giving the upper curves. The horizontal lines indicate (a) Dashed lines ‐ the Southall et al. (2007) threshold for PTS in gray seals (203 dB) and harbor porpoise (215 dB) when exposed to nonpulsed sound and (b) Solid lines ‐ thresholds for PTS for use with A‐weighting. The latter are 95 dB above the threshold of hearing (Heathershaw et al., 2001), which equates to 166 dB for gray seals and 175 dB for harbor porpoise at 1 kHz. Gray shading gives a 95% prediction interval, that is, the central 95% of SELs calculated for simulated animals. Note nonlinear *x*‐axis for display, and sound levels are dB re 1 μPa2/s

The choice of weighting scheme, even in combination with its associated threshold, had a marked effect on the proportion of the simulated population estimated to experience PTS (Figure 2 and Table 2). Regardless of the period over which agents were exposed to noise, there were large (tens of dB) differences for both species between the estimates of SEL made using the two different weightings (Figure 3). Although different thresholds for PTS are associated with these weightings, they do not make these weighting schemes equivalent, as measured by the proportion of the population estimated to experience PTS. This is shown in Figure 3 by the 95% prediction ellipses (the central 95% of SELs for the simulated population) in relation to their PTS thresholds.

**Table 2:** Percentage of simulated animals that exceed a PTS threshold over time

	Weighting	PTS threshold (dB)	Scenario length (hr)							
			1	6	12	24	48	96	168	240
Gray seal	A	166	0.0	0.0	0.0	0.0	0.0	0.0	0.0	0.0
	M	203	0.14	2.55	5.58	7.55	9.75	11.28	12.28	13.78
Harbor porpoise	A	175	0.0	0.0	0.0	0.0	0.0	0.0	0.0	0.0
	M	215	0.0	0.0	0.0	0.0	0.0	0.0	0.0	0.0

SELs are calculated using either an audiogram weighting (A) or the M‐weighting (M) of Southall et al. (2007). Thresholds for PTS are those recommended in Southall et al. (2007) in the case of M‐weightings and “audiogram appropriate” figures from Heathershaw et al. (2001) for A‐weighting.

The practical effect of the choice of weighting, and therefore PTS threshold, was very marked (Table 2). No gray seal agents were predicted to experience PTS when A‐weightings were used. However, 2.6% of gray seal agents were predicted to experience PTS after 6 hr of exposure when M‐weightings were used, and 13.8% were predicted to experience PTS after 10 days of exposure.

### 3.2 Responsive movement

**Figure 4:**
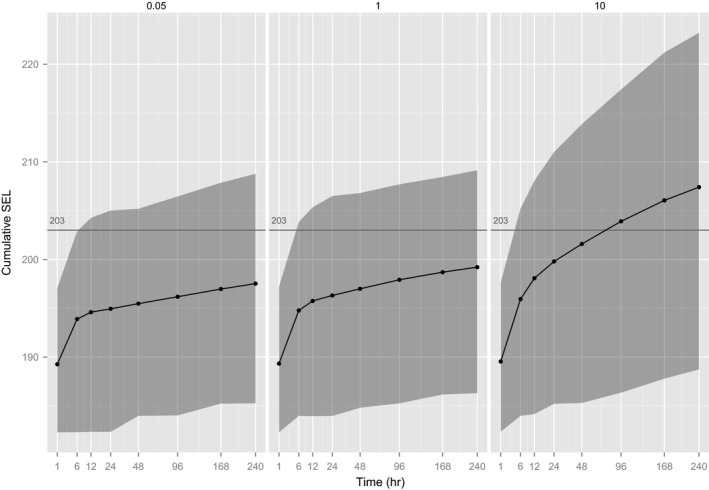
The effect of different degrees of responsive movement by gray seals on SEL. A standard deviation of 10 results in directionless movement; a standard deviation of 0.05 results in marked avoidance of the source. The horizontal line is the threshold (203 dB) for PTS suggested by Southall et al. (2007) for pinnipeds exposed to nonpulsed sound. Gray shading gives a 95% prediction interval, that is, the central 95% of SELs calculated for simulated animals. Note nonlinear *x*‐axis for display, and sound levels are dB re 1 μPa2/s

The magnitude and directionality of the avoidance responses also affected the estimated SEL (Figure 4). The effect depended on the duration of the scenario. The interval is widest when *SD* = 10, which represents a situation in which there is effectively no response to sound. After 1 day of exposure, the average difference in the SEL for agents that showed a directionless response was about 5 dB higher than for agents that showed very directed movement. After 10 days, the difference was in the order of 10 dB.

### 3.3 Constrained movement

**Figure 5:**
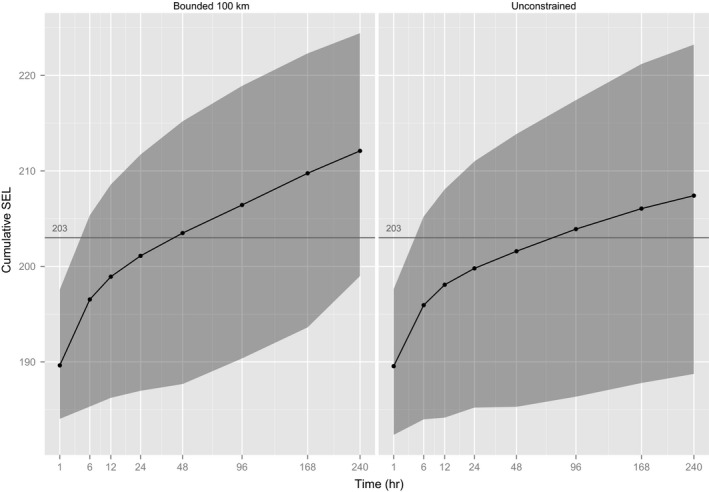
The effect of constraining movement of gray seals to within 100 km of the sound source on long‐term SEL. The horizontal line is the threshold (203 dB) for PTS suggested by Southall et al. (2007) for pinnipeds exposed to nonpulsed sound. Gray shading gives a 95% prediction interval, that is, the central 95% of SELs calculated for simulated animals. Note nonlinear *x*‐axis for display, and sound levels are dB re 1 μPa2/s. Animals are specified to have low levels of responsive movement

**Figure 6:**
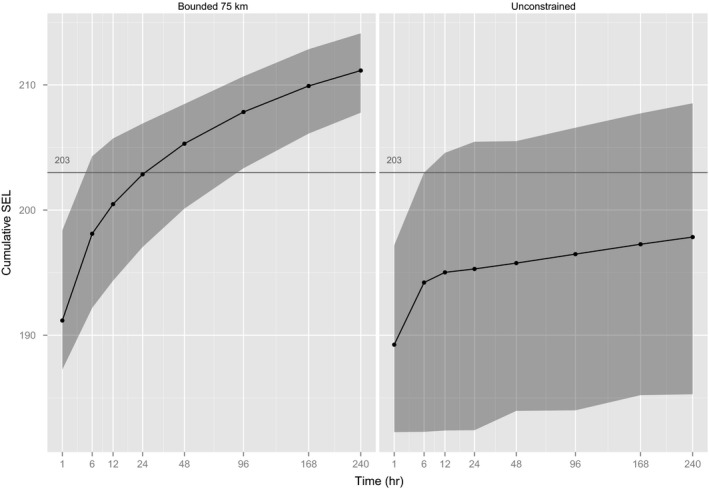
The effect of constraining movement of gray seals to within 75 km of the sound source on long‐term SEL. The horizontal line is the threshold (203 dB) for PTS suggested by Southall et al. (2007) for pinnipeds exposed to nonpulsed sound. Gray shading gives a 95% prediction interval, that is, the central 95% of SELs calculated for simulated animals. Note nonlinear *x*‐axis for display, and sound levels are dB re 1 μPa2/s. Animals have been specified to have a moderate level of responsive movement

The effect of a physical constraint on SEL was less than the simple effect of weighting scheme or directed movement (2 dB more after 1 day of exposure and 5 dB more after 10 days), as seen when agents were constrained to stay within 100 km of the source (Figure 5, no aversion). However, the effect of constraint becomes more marked if combined with directed movement (8 dB more after 1 day and 15 dB more after 10 days), as seen when constrained to stay within 75 km of the source (Figure 6, moderate aversion).

## 4 Discussion

SAFESIMM was used to investigate the probability that individuals of two marine mammal species will experience a physical effect (PTS) under a range of different scenarios and to illustrate the level of uncertainty associated with these predictions.

Simulation frameworks offer a powerful way to explore, understand, and estimate effects of cumulative sound exposure on marine mammals. However, important but subjective assumptions that can dramatically alter their predictions may be hidden within them. For example, they may, as illustrated here, be underpinned by different auditory weighting functions. These different assumptions may result in different recommendations being made to managers about the sound exposure levels that will exceed allowable harm limits; in this example, the proportion of the local population estimated to experience PTS. This difference is largely a consequence of the combination of the weighting scheme and injury thresholds/functions that are applied; although more subtly, response to sound is also a function of SELs. However, while there is an unambiguous pairing of weightings and thresholds in Southall et al. (2007), there are no similar standard recommendations for use with A‐weightings. If the weighting approach is not mandated by regulators, developers can provide very different risk assessments for exactly the same sound exposure scenario depending on which simulation framework they use.

Our results also highlight that the sensitivity of results to certain assumptions depends on the timescale over which animals are exposed to anthropogenic noise. A great deal of effort has, and can be, expended on accommodating fine‐scale movement behaviors of agents within the models. The effort is both at a programming level and subsequent provision of parameter estimates. We have varied one such parameter, avoidance, which is arguably the most relevant in terms of the accumulation of sound exposure. This is relatively unimportant for short‐term (<12 hr) exposures, but becomes more important as the duration of exposure increases. We can infer from this that finer‐scale details of 3D animal movement (such as pitching or yawing) are likely to have an even smaller effect on cumulative sound exposure for short scenarios.

Predictions for longer‐term scenarios are more dependent on the assumed movement models, and any boundaries imposed on that movement. These could either be hard boundaries, such as land, or virtual boundaries such as those imposed by site fidelity where individuals have a strong preference to stay within a restricted area. We approximated this kind of site fidelity by limiting the distance animals could move away from the source. In the long term, an animal's acceptance of sound exposure and its decision to remain within a preferred environment will affect its cumulative exposure levels. However, there is little information on how animals respond in the longer term to sound exposure (Morton & Symonds, 2002; Thompson et al., 2013). For example, in general, we do not know whether they leave an area where they are exposed to noise and never return, if they return within some period of time, or if they remain in the vicinity of the noise source, despite disturbance. In reality, these responses are likely to be context specific. Given these uncertainties, we need to be aware of the sensitivity of long‐term simulations to the assumptions that underpin the treatment of movement, because long‐term predictions may simply reflect subjective decisions about these assumptions.

We found that predictions of SELs over long durations were primarily constrained by limitations in knowledge (i.e., the ability to parameterize the movement models with empirical data). The proximate cause of this lack of data is probably the result of logistical constraints on long‐term deployment of tags on marine mammals (Johnson, de Soto, & Madsen, 2009), but its ultimate cause may be a legacy of the fact that research priorities have been driven by the needs to predict the short‐term, acute impacts of military sonar on acoustically sensitive marine mammals. However, long‐term data are needed to assess and mitigate the impacts of offshore renewable energy construction on marine mammals. This is a relatively new industry and, to date, sufficient data have not been collected to support these new impact assessments.

The assumption that had the greatest influence on the estimates of the proportion of agents that experienced PTS was the choice of weighting scheme. However, in our view, at present published data are insufficient to justify the choice of one weighting scheme over another. Therefore, regulators and their scientific advisors need to be aware that the choice of weighting scheme is likely to have a profound effect on the predictions made using simulation frameworks, and greater transparency about the assumptions that are embedded in these frameworks is required. This serves as an important reminder that managers and policymakers are obliged to understand these assumptions and make decisions about how much risk they are willing to tolerate.

## Conflict of interest

None declared.
